# Proline as a Sparker Metabolite of Oxidative Metabolism during the Flight of the Bumblebee, *Bombus impatiens*

**DOI:** 10.3390/metabo11080511

**Published:** 2021-08-04

**Authors:** Nadia Stec, Ammar Saleem, Charles-A. Darveau

**Affiliations:** 1 Department of Biology, University of Ottawa, Ottawa, ON K1N 6N5, Canada; nstec029@uottawa.ca; 2 Center for Advanced Research in Environmental Genomics, Laboratory for the Analysis of Natural and Synthetic Environmental Toxins, University of Ottawa, Ottawa, ON K1N 6N5, Canada; asaleem@uottawa.ca

**Keywords:** proline, insect, flight, metabolism, carbohydrates, glycolysis, TCA cycle, hymenopterans

## Abstract

Several insect species use the amino acid proline as a major energy substrate. Although initially thought to be limited to blood-feeding dipterans, studies have revealed this capability is more widespread. Recent work with isolated flight muscle showed that the bumblebee *Bombus impatiens* can oxidize proline at a high rate. However, its role as a metabolic fuel to power flight is unclear. To elucidate the extent to which proline is oxidized to power flight and how its contribution changes during flight, we profiled 14 metabolites central to energy and proline metabolism at key time points in flight muscle and abdominal tissues. Ultra-high performance liquid chromatography-electrospray ionization-quadrupole time of flight mass spectrometry (UPLC-ESI-QTOF MS) analysis revealed that proline is likely used as a sparker metabolite of the tricarboxylic acid cycle at the onset of flight, whereby it supplements the intermediates of the cycle. Carbohydrates are the major energy substrates, which is evidenced by marked decreases in abdominal glycogen stores and a lack of alanine accumulation to replenish flight muscle proline. The time course of fuel stores and metabolites changes during flight highlights homeostatic regulation of energy substrates and patterns of changes in metabolic intermediates within pathways. This study clarifies the role of proline and carbohydrate metabolism during flight in hymenopterans, such as *B. impatiens*.

## 1. Introduction

Animals power their locomotion using various energy substrates, mostly carbohydrates and lipids, but sometimes favoring amino acids. Flying insects also exhibit multiple strategies. For example, locusts initially use carbohydrates as their main fuel, but eventually switch to the high energy content of lipids for long flights [1]. Other insects use amino acids as a source of energy, such as the blood-feeding tsetse fly, *Glossina morsitans*, that fuels its flight using proline almost exclusively [2,3]. In other insect species, proline can be oxidized in combination with other substrates [4,5]. Proline oxidation can be used to augment tricarboxylic acid (TCA) cycle intermediates required for acetyl-CoA oxidation [2,6], thereby serving as a so-called sparker of mitochondrial metabolism. For example, the blowfly *Phormia regina* uses proline as a sparker to supplement TCA cycle intermediates at the start of flight [7], and when their mitochondria are supplied with proline and pyruvate, TCA cycle intermediate content increases, thereby increasing pathway flux [8]. In the Colorado potato beetle [9] and African fruit beetle [10], proline can be used simultaneously with carbohydrates over prolonged periods. Although used to various extents and over different timelines among species that use it as main fuel or as a sparker of the TCA cycle, proline oxidation appears to be a common metabolic feature of diverse insect groups.

Proline oxidation involves only a few enzyme-catalyzed reactions initiated by proline dehydrogenase, ultimately producing glutamate. Although insects can completely oxidize proline by glutamate dehydrogenase and subsequent reactions, partial proline oxidation is commonly observed where glutamate and pyruvate are substrates for alanine aminotransferase (AAT), producing α-ketoglutarate and alanine [10]. The produced alanine is used to resynthesize proline in the fat body, making the process an osmotically neutral and nitrogen-waste-free pathway [11]. The high solubility of proline and its high concentration in both flight muscle and hemolymph make it readily available as a fuel, and it does not require specific carrier proteins [12,13]. Proline can also act as a carbon shuttling molecule between lipid stores in the fat body and muscles [13,14]. Proline appears to undergo partial oxidation in most insects when used as a sparker, exclusively, or in combination with carbohydrates [6]. Evidently, proline can be more than just a dietary opportunity for insects.

The honeybee *Apis mellifera* is often used to exemplify bees as a group. Honeybees rely almost exclusively on carbohydrates to fuel their activity since plant nectars are rich in sugars [15]. The use of proline as a metabolic fuel for flight in honeybees has also been investigated, though it was estimated that only 0.1% of energy produced is generated using proline [16]. The concentration of proline in flown honeybees was significantly lower than in resting bees, but the amount metabolized is low compared to carbohydrates [17]. Similarly, decreases in proline are seen in foragers returning from foraging flights [18]. Honeybees may not have the capacity to use proline as a major fuel because their flight muscles do not contain sufficiently high activity of proline dehydrogenase [19]. Circulatory carbohydrates and stored glycogen are the fuel of choice because they can be metabolized readily by flight muscles [3]. Nevertheless, proline metabolism has not been well documented in other Hymenopteran species.

Nectars of pollinator-attracting plants are not only rich in sugars, but contain a substantial amount of proline relative to other amino acids. Proline was proposed to act as a metabolic reward to attract pollinators [15]. Recently, it was shown that isolated flight muscle fibers of several hymenopterans exhibit a high capacity to oxidize proline; research showed that a bumblebee (*Bombus impatiens*) and a wasp (*Vespula vulgaris*) could double the oxygen consumption rate by adding proline to fibers oxidizing carbohydrate-derived substrates, while the *A. mellifera* showed no detectable increase [20]. This study also showed that bumblebees’ flight muscles do not have the capacity to power flight using lipids as shown for other bee species [20]. Despite the high capacity to oxidize proline, it is unclear if *B. impatiens* uses proline as an energy substrate for flight. The capacity to use proline as a way to enhance the oxidation of carbohydrates has been documented previously in both dipterans [5,21,22,23,24] and coleopterans [2,13,25,26]. Thus, it is possible that bumblebees use proline as a fuel in combination with carbohydrates, which may be a feature of many hymenopteran species.

Founding studies profiled metabolites during the flight of blowflies for an hour, measuring changes in intermediates of carbohydrate and proline metabolism [27,28]. These experiments demonstrate how metabolites and intermediates change during the first seconds to minutes of the flight of *P. regina*, highlighting the sparker role of proline early in the dipteran’s transition from rest to flight. In coleopterans using both carbohydrates and proline during flight, muscle proline is used as the main substrate at the onset of flight, also within seconds or minutes, and alanine accumulates as an end product [10]. Next, abdominal glycogen content decreases due to increased demands of carbohydrate metabolism. Finally, metabolite levels stabilize after around eight minutes of flight, and flight performance is unchanged. By observing fluctuations in metabolites, it is possible to gain insight into metabolic pathways involved during flight.

In this study, we measured changes in metabolite content during the progression of flight to assess (i) the extent to which proline is oxidized to power flight, and (ii) how the contribution of proline changes throughout prolonged flight. Metabolites of the proline catabolic pathway, glycolytic pathway, and TCA cycle were profiled at several time points during flight using UPLC-ESI-QTOF MS. Fluctuations of metabolite contents in vivo were expected to complement the in vitro findings of Teulier et al. [20]. We show significant changes in metabolite concentration during the progression of flight, mainly a decrease in thoracic proline content over the first few minutes of flight without accumulation in alanine, suggesting that proline is used to supplement intermediates of the TCA cycle, and not as a fuel used for sustained flight. Profiling metabolites involved in energy metabolism further shows the central role of carbohydrates and changes in pathway metabolites steady state during the progression of a 30-min flight.

## 2. Results

### 2.1. Metabolites Profile Changes

**Figure 1:**
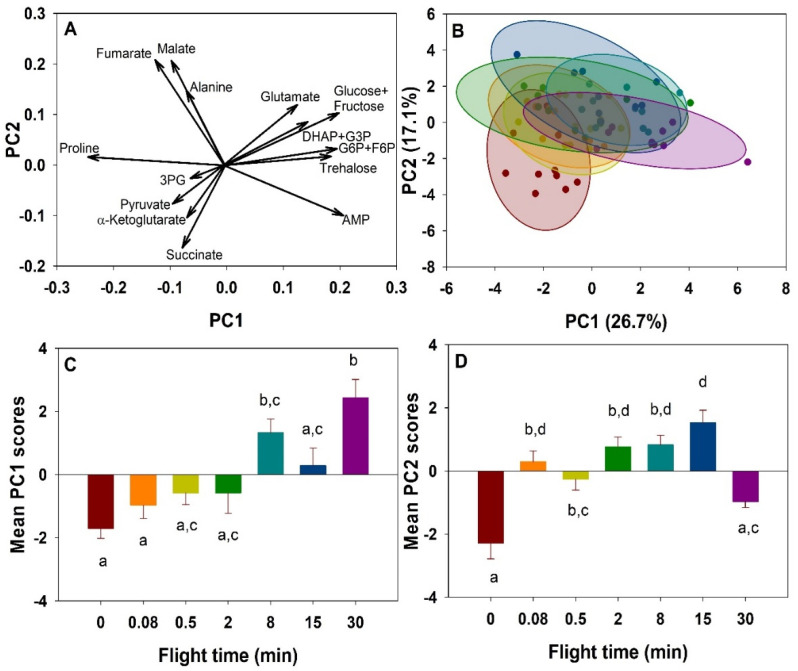
Changes in energy metabolism metabolite profiles during a 30-min flight in the thorax of the bumblebee *Bombus impatiens*. PCA plots for thoracic metabolite data. (**A)**. Biplot of the thoracic metabolites. (**B**). Scores plot of collective metabolite intensities of individual bees at each time point during flight. Colors of clusters correspond to flight times (red = 0 min, orange = 5 s, yellow = 30 s, green = 2 min, light blue = 8 min, dark blue = 15 min, purple = 30 min). (**C**). Changes in mean PC1 scores over time (*p* < 0.001). (**D**). Changes in mean PC2 scores over time (*p* < 0.001). Columns that do not share a letter are significantly different.

Overall changes in metabolites during the progression of flight were summarized using a principal component analysis (PCA). Fourteen metabolites detected in the thorax were reduced to the first two principal components, together accounting for 43.8% of the variation. In the biplot (Figure 1A), PC1 is dominated by trehalose, glucose-6-phosphate (G6P)+fructose-6-phosphate (F6P), as well as proline in the opposite direction. Other metabolites vary in the same direction as trehalose and G6P+F6P, namely glutamate, dihydroxyacetone phosphate (DHAP)+glyceraldehyde-3-phosphate (G3P), glucose+fructose, and adenosine monophosphate (AMP). PC2 appears to be dominated mainly by fumarate, alanine, and malate in the positive direction, while α-ketoglutarate, pyruvate and succinate contribute most to variation in the opposite direction. Individual metabolic profiles represented and grouped by flight time show a shift in the clusters over time, indicating an overall profile change during the progression of flight (Figure 1B). PC1 values increase significantly over time (F_6,63_ = 8.615, *p* < 0.001, Figure 1C), while PC2 increased significantly from rest to most flight times (F_6,63_ = 13.319, *p* < 0.001, Figure 1D).

### 2.2. Proline Metabolism

**Figure 2:**
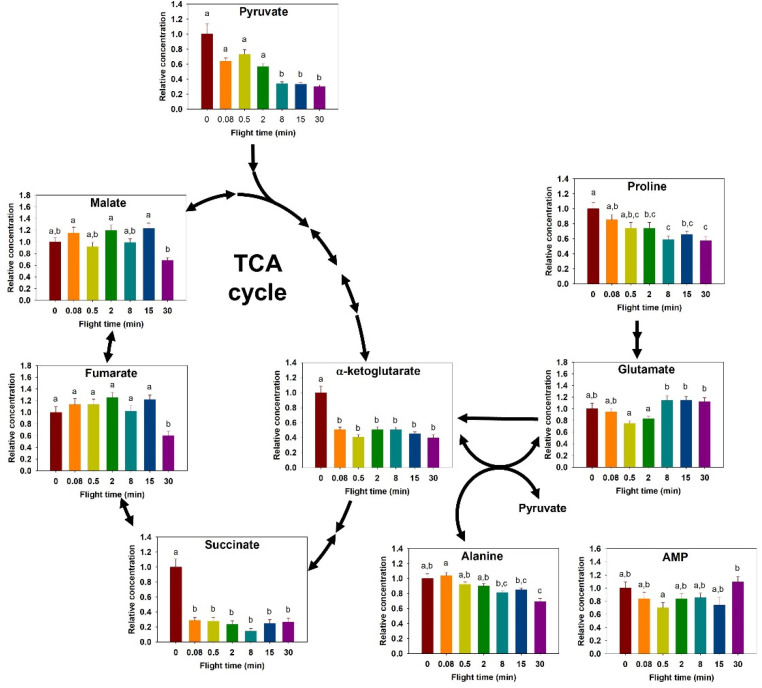
Relative change in tricarboxylic acid cycle (TCA) metabolism intermediates during the progression of a 30-min flight in the thorax of the bumblebee *Bombus impatiens*. Colors correspond to flight times (red = 0 min, orange = 5 s, yellow = 30 s, green = 2 min, light blue = 8 min, dark blue = 15 min, purple = 30 min). Columns that do not share a letter are significantly different (*p* < 0.05).

Thoracic proline concentration decreased with greater flight time (F_6,63_ = 6.464, *p* < 0.001, Figure 2). Pair-wise comparisons showed a significantly lower concentration after two minutes of flight, and a total decrease of 44% in proline relative concentration after 30 min of flight. Alanine concentration also decreased during flight (F_6,63_ = 8.94, *p* < 0.001, Figure 2). Pairwise comparisons revealed a decrease in concentration, occurring between the shortest time points and those exceeding 8 min (*p* < 0.03). The largest change was a 33% decrease in concentration between 0.08 min and 30 min. Glutamate relative content also changed over time (F_6,63_ = 6.137, *p* < 0.001, Figure 2). Pairwise comparisons showed the glutamate content increased significantly between the shortest flight times of 0.5 and 2 min, and the 8, 15, and 30 min flights (*p* < 0.05).

### 2.3. Carbohydrate Metabolism

**Figure 3:**
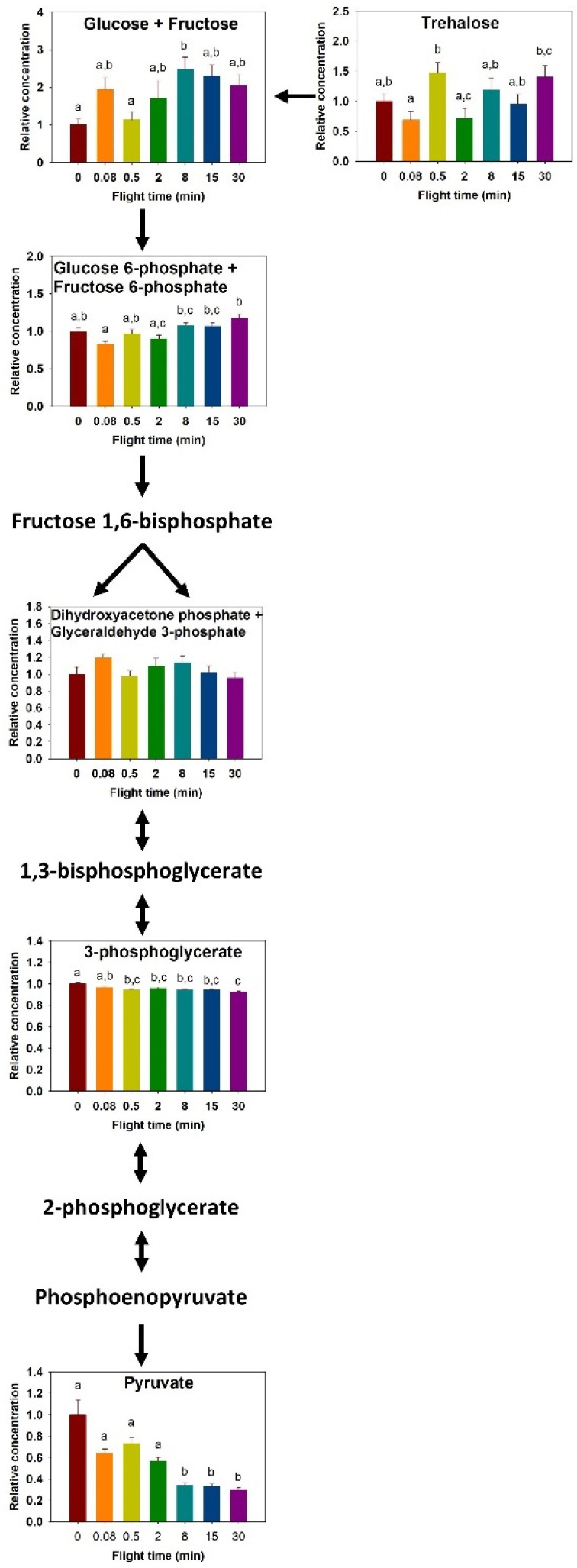
Relative change in carbohydrate metabolism intermediates during the progression of a 30-min flight in the thorax of the bumblebee *Bombus impatiens*. Colors correspond to flight times (red = 0 min, orange = 5 s, yellow = 30 s, green = 2 min, light blue = 8 min, dark blue = 15 min, purple = 30 min). Columns that do not share a letter are significantly different (*p* < 0.05).

The concentration of thoracic trehalose changed throughout 30 min of flight (F_6,63_ = 3.703, *p* = 0.003, Figure 3). Increases occurred from 0.08 to 0.5 min (*p* = 0.017) and 0.08 to 30 min (*p* = 0.049). Glucose+fructose increased over time (F_6,63_ = 3.322, *p* = 0.007, Figure 3), where the lowest concentrations observed at 0 and 0.5 min of flight time were significantly different than the highest content observed at 8 min (*p* < 0.05).

Glycolytic intermediates detected in tissue samples included G6P+F6P, DHAP+G3P, 3PG, and pyruvate. G6P+F6P changed significantly (F_6,63_ = 5.717, *p* < 0.001, Figure 3), with increases from 0.08 to 8, 15, and 30 min (*p* = 0.010, 0.012, and *p* < 0.001, respectively), and from 2 to 30 min (*p* = 0.004). Although the concentration of DHAP+G3P did not change significantly during the progression of flight, its pattern of change is strikingly similar to glucose+fructose, and so is the trehalose and G6P+F6P pair, in agreement with the PCA (Figure 1). Metabolites for the second half of glycolysis show distinct changes in concentrations, where 3PG decreased slightly from rest and 0.5 min of flight and beyond (F_6,573_ = 9.012, *p* < 0.001, Figure 3). Pyruvate changes in concentration were more substantial (F_6,50_ = 13.69, *p* < 0.001, Figure 3), where it decreased by 70% between rest and 30 min of flight; pyruvate content was lower than all shorter flight times after 8 min of flight, and remained low (*p* < 0.05).

### 2.4. TCA Cycle

Detected TCA cycle intermediates included α-ketoglutarate, succinate, fumarate, and malate. In the thorax, α-ketoglutarate and succinate concentrations changed during flight (α-ketoglutarate: F_6,63_ = 20.091, *p* < 0.001; succinate: F_6,60_ = 24.512, *p* < 0.001, Figure 2). For both α-ketoglutarate and succinate, the concentration at rest was markedly higher than at all other times (*p* ≤ 0.001, Figure 2), corresponding to a 60 and 73% decrease from rest after 30 min of flight, respectively. Fumarate and malate showed similar differences (fumarate: F_6,62_ = 6.051, *p* < 0.001; malate: F_6,63_ = 5.694, *p* < 0.001, Figure 2), where a sharp decline occurred after 30 min of flight. Finally, AMP appeared to be changing (F_6,63_ = 2.732, *p* = 0.04, Figure 2), but differences were detected only between 0.5 min to 30 min (*p* = 0.044).

### 2.5. Abdominal Metabolites Content

**Figure 4:**
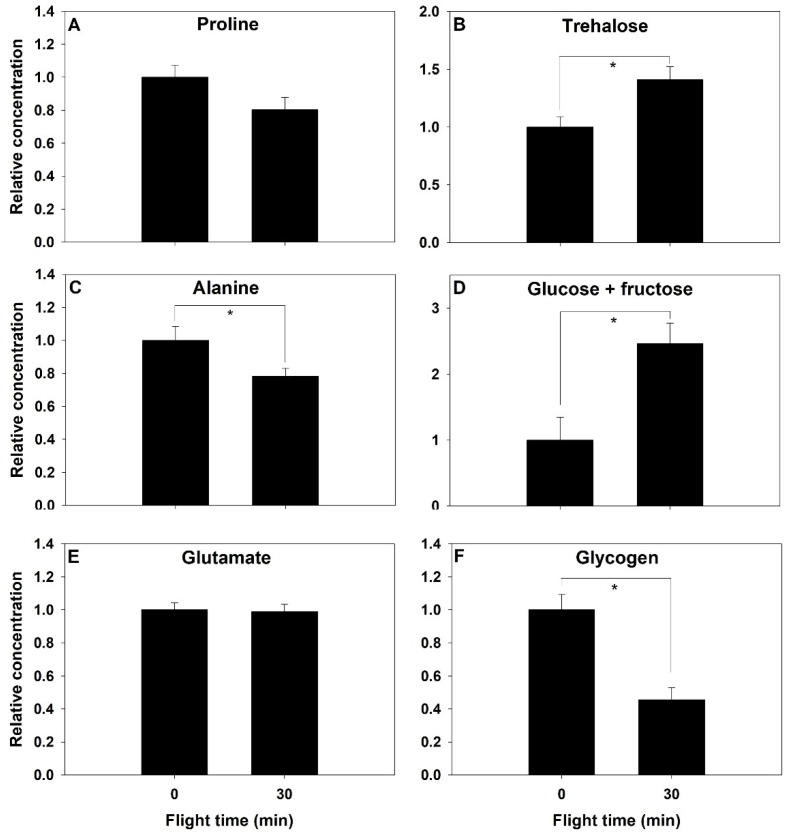
Relative change in energy metabolism intermediates (**A**–**F**) before and after a 30-min flight in the abdomen of the bumblebee *Bombus impatiens*. Asterisk indicates a significant difference (*p* < 0.05).

Metabolites content of the abdomen were measured at rest and after 30 min of flight time. Proline concentration did not change significantly after 30 min of flight (F_1,18_ = 3.59, *p* = 0.074, Figure 4A), but alanine showed a significant decrease of similar magnitude of 22% (F_1,18_ = 5.00, *p* = 0.038, Figure 4C). Glutamate did not change in the abdomen (*p* > 0.05, Figure 4E). Abdominal trehalose concentration increased by 41% after 30 min (F_1,18_ = 7.552, *p* = 0.013, Figure 4B), and glucose+fructose more than doubled during this period (F_1,18_ = 9.765, *p* = 0.006, Figure 4D). Glycogen concentration decreased by more than half during the 30 min of flight (F_1,8_ = 8.804, *p* = 0.002, Figure 4F).

## 3. Discussion

Several insect species can use the amino acid proline as a metabolic fuel, but the evolutionary origin and the adaptive role of this metabolic diversity remains unclear. In the order Hymenoptera, honeybees’ flight muscle fibers can oxidize carbohydrate-derived substrates almost exclusively, while sister species, such as the bumblebee (*B. impatiens*), can also oxidize proline at high rates [20]. The capacity to oxidize the amino acid proline may be linked to the ability to use it as a fuel for flight. We show that changes in metabolites content profile of energy metabolism pathways during the progression of flight are clearly detected. Proline content is depleted over the first few minutes of flight, but the absence of alanine accumulation indicates that it may not be a substantial and sustainable fuel for flight in bumblebees. Nevertheless, changes in proline content follow a similar timeline to species using proline and carbohydrates as fuel during the initiation of flight, where large changes occur in the first 2 to 10 min of flight [10,28], suggesting that it plays an important role in fueling oxidative phosphorylation early in flight. Metabolic profiling also revealed that metabolite content is tightly regulated, but changes over shorter flight times are substantial, where metabolic transitions are the greatest. Proline appears to be a fuel contributing to the early phases of flight in bumblebees.

Insect species use proline as (1) a single metabolic fuel, (2) transiently as a sparker of the TCA cycle, or (3) more extensively as a co-substrate with carbohydrates [10]. We hypothesized that, in bumblebees, proline is used as a co-substrate with carbohydrates during flight, since isolated flight muscle mitochondria can oxidize it at a high rate and proline is found in plant nectar [15,20]. Species that use proline as a co-substrate with carbohydrates, such as the coleopteran Colorado potato beetle, show a constant decrease in thoracic proline concentration over the first 10 min of flight, accompanied by a proportional increase in alanine [9,26]. The African fruit beetle also shows a decrease in thoracic proline concentration and corresponding alanine accumulation upon the initiation of flight, but these changes occurred over shorter periods, indicating that proline contributes substantially to oxidative metabolism during the first two minutes [10]. We saw a gradual decrease in thoracic proline over the first eight minutes of flight, similar to the temporal patterns observed for beetle species. However, alanine did not accumulate in a reciprocal manner (Figure 2).

Bumblebees use proline during the first couple minutes of flight, but appear to oxidize it completely because it does not lead to alanine accumulation during that period. Partial proline oxidation involves the reaction catalyzed by the enzyme AAT leading to alanine accumulation in the thorax. A study conducted on isolated mitochondria of the Japanese beetle *Popillia japonica* has shown that the oxidation of proline was associated with more NH_3_ accumulation than alanine, suggesting that some species favor complete over partial oxidation [29]. *Bombus impatiens* appears to use this strategy, as no alanine accumulated while proline decreased (Figure 2), but we did not include NH_3_ measurements in our study to support it. The most widely documented pathway involving proline oxidation in insects is by far its partial oxidation, where alanine accumulates in proportion to proline degradation. Nonetheless, other possibilities involving other reactions, such as aspartate aminotransferase, have been considered [19]. Furthermore, a portion of proline remains completely oxidized in species fueling flight solely using proline, such as the tsetse fly. The activity of both AAT and glutamate dehydrogenase (GDH) is high in many insects’ flight muscle (reviewed in [30]).

Further evidence that bumblebees do not use proline as a main fuel for flight comes from abdominal concentrations in proline and alanine. In species that use proline as a major fuel to power flight (such as the African fruit beetle), decreases in abdominal proline content have been reported, while alanine concentration increased following 15 min of flight [10]. These changes are due to proline being mobilized from the abdomen to the flight muscle to sustain flight, while alanine from the flight muscle is transported and accumulated in the abdomen. Abdominal alanine is used to resynthesize proline in the fat body. This process is controlled by hormonal regulation by the adipokinetic hormone family of neuropeptides through the addition of two carbons from stored triglycerides [31]. We did not observe the accumulation of alanine in the abdomen, which indicates that proline use is not compensated by its synthesis in the fat body, suggesting that its use is only transient and short term.

Changes in energy production pathway metabolite content are due to the balance between their use and production. Glutamate is an intermediate metabolite of the proline oxidation pathway. Its concentration is dependent on its production rate that is a function of proline degradation rate. Its use is a function of the integration of α-ketoglutarate in the TCA cycle via the reaction catalyzed by AAT and/or GDH. The observed decline in glutamate content over the first two minutes of flight indicates a transition in the pathway’s flux where its use is greater than its production rate, which is what was also observed in the blowfly [28]. No further changes were observed beyond two minutes of flight in both the bumblebee and the blowfly, suggesting that a new steady state is reached or that the pathway is no longer active; the absence of decline in proline content suggests that the latter is more likely.

The importance of carbohydrates in fueling flight is reflected in the changes in concentrations of metabolic fuels and pathway intermediates. The extent of glycogen store use is a function of the availability of dietary carbohydrates where glycogen stores are depleted more rapidly in unfed insects [32,33]. Thoracic trehalose and glucose concentrations show more variability upon the initiation of flight, likely reflecting the balance between their use and synthesis. Some species, such as the flying cockroach, show a gradual decrease in thoracic muscle trehalose during the progression of a 15 min flight, but their glucose content shows an initial increase after 5 min, followed by a decrease [32]. In mosquitoes, which power their flight using carbohydrates, no clear change in trehalose content was observed, while glucose content decreased following prolonged flight [33]. In the blowfly, trehalose rapidly decreases within the first 30 s, and then the changes in concentration are reduced considerably. In contrast, glucose shows a sharp increase within the first minutes of flight, which then stabilizes to constant levels for the next hour [28]. We observed no decrease in thoracic trehalose concentration during the progression of flight (Figure 3), which contrasts with the findings in many insect species. It is possible that the lack of decrease in trehalose content reflects the high rate of trehalose synthesis by the fat body, in turn impacting and maintaining the concentration of its cleavage product glucose. Blatt and Roces [34] found that honeybees exposed to low temperatures could not maintain hemolymph trehalose levels because their metabolic rate increased to match that of flight metabolism. They inferred that trehalose synthesis rate from the fat body was sufficient to maintain hemolymph trehalose content constant in low to moderate metabolic rate. We observed a significant decrease in abdominal glycogen stores and a corresponding increase in trehalose and glucose+fructose concentrations of the abdomen (Figure 4), in agreement with the synthesis of circulatory carbohydrates by the fat body.

Glycolytic metabolites show two main patterns of change during continuous flight. There is an apparent difference between the initial steps of the glycolytic pathway and those occurring in the latter half, where we found that G6P and F6P increased by the 30-min mark, whereas 3PG and pyruvate decreased (Figure 3). In the blowfly, a transient increase in G6P occurs within 30 s [28], as well as in the locust [35]. Aging flight muscle of *Manduca sexta* moths has greater glycolytic demand and demonstrates increased pyruvate, 3PG, G6P, and F6P [36]. There are no consistent changes in glycolytic intermediates, although we observe an apparent accumulation of metabolites above the regulatory enzyme phosphofructokinase and decrease in downstream metabolites.

TCA cycle intermediates also show two main patterns of metabolite changes. The concentration of α-ketoglutarate and succinate show an initial sharp decrease upon the initiation of flight, followed by a new steady state for the remainder of the 30-min flight, while fumarate and malate decrease at the end of the flight period rather than the beginning (Figure 2). A similar steady state of TCA cycle intermediates is observed in the blowfly, but, in this species, malate alone shows a transient increase at the onset of flight [28]. Metabolomic studies in fruit flies demonstrate that, when succinate dehydrogenase (SDH) function is disrupted, there is an accumulation of succinate and reduction of downstream TCA cycle intermediates [37]. The same study states that TCA cycle intermediates are rescued at the point of α-ketoglutarate, likely from anaplerotic input, such as the deamination of glutamate. We observed a sharp decrease within 10 s of flight in succinate and α-ketoglutarate, followed by a plateau (Figure 2). Normally functioning SDH appears to regulate the latter half of the TCA pathway, as succinate is rapidly oxidized in order to produce fumarate and, subsequently, malate, which remained steady throughout bumblebee flight. The decrease we observed in α-ketoglutarate (Figure 2) might be attributed to large quantities required for pyruvate oxidation and ATP production at the start of flight. Its steady state may be maintained by glutamate use via AAT and/or GDH.

The metabolites changes seen in *B. impatiens* correspond to changes observed in species that use both carbohydrate and proline to power flight. The snapshots of metabolites profile changes throughout flight support the role of proline as a sparker that supplements the TCA cycle. We reached this conclusion because (1) bumblebee mitochondria show a high capacity to oxidize proline [20], (2) the timeline of thoracic proline decline within the first few minutes of flight is similar to species using proline as a sparker of the TCA cycle or co-substrate with carbohydrates, and (3) the decrease in proline at the onset of flight, with no concomitant increase in alanine, indicates that it is not sustainable long term. Further measures of amino acid catabolism, including ammonia and other products such as aspartate, would help confirm this. It is also possible that higher concentrations of circulatory and tissue proline coming from the diet may substantially increase its contribution to energy production and should be considered. The changes in carbohydrates and fuel stores also reflect their central contributions. Glycogen content decreased in the abdomen with a reciprocal increase in trehalose, indicating homeostatic mechanisms supplementing trehalose supply to the flight muscles. Together, these findings indicate that carbohydrates are the main fuel used to generate ATP for sustained flight, with proline likely supplementing TCA cycle intermediates at the onset of flight and meeting the large increase in flux rates through energy metabolism pathways. Nevertheless, the ability to oxidize proline is not present in all hymenopterans [20]. Further insight is required to understand how and why it evolved in species such as bumblebees.

## 4. Materials and Methods

### 4.1. Flight Experiments

*Bombus impatiens* colonies (Biobest, Leamington, ON, Canada) containing one queen and 20 workers were maintained at 22 °C. Bees were fed ad libitum a diet of pollen and nectar solution. Adult worker bumblebees were used for all experiments. Bees were captured, cooled down in the refrigerator, and an insect pin was fixed to the top of the thorax, between the wings, using UV-cured resin (Solarez, Vista, CA, USA). Bees were tethered to a 30 cm diameter flight mill and allowed to adjust to a room temperature of 22 °C, at which flights were conducted. Flight was stimulated by the tarsal reflex and presenting visual flow to the animal. If flight was irregular or discontinuous, the individual was returned to the colony. The duration of continuous flights included 5 and 30 s, and 2, 8, 15, and 30 min, as well as a control group of resting bees that performed no flight. Individuals reached the desired duration at continuous or near-continuous flight where it was successfully reinitiated within 30 s. Bees were immediately freeze-clamped with metal tongs chilled in liquid nitrogen and then dipped in liquid nitrogen to thoroughly freeze the tissues. Frozen bees were stored at −80 °C. A sample size of *n* = 10 individuals were used for all flight durations, with the exception of glycogen measurements where *n* = 5.

### 4.2. Preparation and Detection of Analytical Standards

Analytical standards (Sigma-Aldrich, Oakville, ON, Canada) were prepared for a total of 29 targeted compounds, in order to assess detection of metabolites and sensitivity of our method. We detected 14 metabolites that spanned the following categories: amino acids: proline, alanine, glutamate; carbohydrates: glucose+fructose, trehalose; glycolysis: G6P+F6P, DHAP+G3P, 3-phosphoglycerate (3PG), pyruvate; adenylate: AMP; TCA cycle: α-ketoglutarate, succinate, fumarate, malate. The pairs of metabolites glucose+fructose, G6P+F6P, DHAP+G3P could not be separated and are therefore presented as combined content. Each standard was prepared as a stock at a concentration of 1 mg/mL by dissolving it in a 40:40:20 solvent mixture of methanol, acetonitrile, and water (Fisher Optima LC-MS, Brockville, ON, Canada). Standards were diluted to 0.01, 0.1, 1, 10, and 100 µg/mL. All standards were stored at −80 °C.

### 4.3. Preparation of Samples

Thoraces and abdomens were dissected, weighed, and placed in an individual centrifuge tube and filled with 9 volumes of solvent mixture. Thoraces and abdomens were minced with scissors and homogenized (Polytron PT 1300 D), then sonicated for 3 min using 5 s pulses at 30 s intervals (Sonics Vibra-cell). The homogenate was centrifuged for 15 min at 10,000× *g* and 4 °C. Supernatant was collected and transferred to glass vials on ice. To maximize the extraction of metabolites, another 9 volumes of solvent were added to the pellet. The pellet was sonicated and centrifuged in the same manner. The supernatant was combined with that from the first extraction and stored at −80 °C in glass vials. Abdomen samples were only collected at time points 0 and 30 min.

All concentrations of standards were plated into a 96-well plate (Waters Inc., Milford, MT, USA). Samples were centrifuged again prior to plating: 300 µL of the supernatant of each sample was transferred to individual centrifuge tubes and centrifuged for 15 min at 10,000× *g* and 4 °C. Then, 200 µL of the supernatant was pipetted into a syringe fitted with a syringe-driven filter unit (Millex, 0.45 um, 4 mm diameter, PTFE membrane, Millipore, Billerica, MA, USA). The supernatant was then loaded into the 96-well plate. Plates were sealed and stored at −80 °C until injection.

### 4.4. Ultra-High Performance Liquid Chromatography-Electrospray Ionization-Quadrupole Time of Flight Mass Spectrometry (UPLC-ESI-QTOF MS)

UPLC-ESI-QTOF MS analyses were undertaken on an Acquity UPLC coupled with XevoG2 QTOF system (Waters Inc., Milford, MT, USA). Separations were performed on a BEH C18, 1.7 µm particle size, 2.1 × 100 mm column connected with a VanGuard pre-column, 2.1 × 5 mm. Mobile phase A (water + 0.1% formic acid) and B (acetonitrile + 0.1% formic acid) (Fisher Optima LC-MS, Brockville, ON, Canada) were delivered at a flow rate of 0.8 mL/min at a column temperature of 65 °C, with the sample temperature at 4 °C. Mobile phase A was delivered isocratic 100% 0–3 min, linear gradient 0–20% B 3–5 min, 100% B isocratic 5–6 min. A 5 µL injection was performed through a 10 µL loop, followed by a strong wash of 200 µL (50% acetonitrile + 50% water) and weak wash of 600 µL (10% acetonitrile + 90% water).

QTOF was operated in positive and negative electrospray ionization (ESI) modes. MassLynx software (Version 4.1) was used to acquire high and low energy spectra in MSe ESI+ and MSe ESI- modes within the mass range of 100–1000 Da. Cone voltages were 15 V in both positive and negative modes, while scan time was set at 0.08 s. Lock mass was set with Leucine Enkephalin C12 at 556.2615 Da [M+H]+1 and 554.261 Da [M-H]-1. Source and desolvation temperatures were 150 °C and 500 °C, respectively. Cone gas and desolvation gas (nitrogen) were set at 50 and 1200 L/h. The molecular ions were acquired at low fragmentation (6 V), and the product ions at high fragmentation (20–40 V). A mass accuracy threshold of 5 PPM and an ion intensity threshold of 1000 was used as criteria for the identification and detection of target compounds, respectively. A few samples did not meet this threshold for pyruvate, 3PG, and fumarate and were not included in the analyses.

### 4.5. Determination of Glycogen Content in Abdomens

Glycogen contents of individual bumblebee abdomens used a protocol adapted from other studies on insects [38,39,40,41]. Briefly, abdomens were separated, weighed, and homogenized in a centrifuge tube in a mixture of 200 µL 2% sodium sulfate solution, 200 µL 70% EtOH, and 300 µL 80% MeOH. The abdomens were minced with scissors and homogenized on ice, then sonicated in 5 s pulses 6 times, with 30 s intervals between pulses. Homogenates were heated for 30 min at 70 °C, and vortexed every 10 min. After cooling at 4 °C for 30 min, the mixture was centrifuged for 10 min at 4 °C and 21,000× *g*. Supernatant was discarded, and the homogenate was resuspended in 80% methanol twice to remove free glucose. A 200 µL sodium sulfite solution and 300 µL EtOH was added and the sample was vortexed, sonicated, then vortexed again. The mixture was centrifuged, and supernatant discarded. The pellet was completely dried at 80 °C, and then 600 µL distilled water was added. After vortexing, the homogenate was left standing for 10 min to allow glycogen to dissolve. The homogenate was vortexed again prior to subsampling, and 120 µL of the fluid was transferred to a clean centrifuge tube and 480 µL of anthrone reagent was added (385 mL 98% sulfuric acid added to 150 mL distilled water, 750 mg anthrone). The mixture was vortexed. Both samples and standards were heated for 17 min at 99 °C, and, after removal from the heating block and cooling, optical density was measured at 625 nm (BioTek Synergy 2 spectrophotometer).

### 4.6. Data Collection and Statistical Analysis

Data collection from the UPLC was performed with MassLynx software (Waters, version 4.1). The relative ion abundance (intensity) of each targeted metabolite was determined from the chromatograph of each analytical standard and thorax and abdomen sample. Signal intensity was converted to concentration using the standard curves generated, except for a few cases that fell outside the range of the standard curve Table S1. For the metabolites glucose/fructose, malate, and α-ketoglutarate signal, intensities were not converted to concentrations as samples fell outside the range of the standard curve. The glutamate standard curve had a poor linear fit and values also were not converted to concentrations. We opted to present changes in concentration during flight relative to resting animals (0 min flight time), as the method was not optimized for improved yield and absolute concentration determination [42,43]. Statistical analysis was completed with Systat 12. Analysis of variance (ANOVA) was conducted, followed by post-hoc tests using Tukey’s honestly significant difference test. In cases where variance was unequal, the Games–Howell test was used for pairwise comparison.

A portion of data analysis was completed using MetaboAnalyst 3.0 (http://www.metaboanalyst.ca, last accessed 20 February 2020), a web-based tool for the processing and analysis of metabolomic data [44]. The data was normalized (normalization by sum) and scaled (autoscaling for column-wise normalization) in order to reduce systematic variance and conduct multivariate analyses. A principal component analysis (PCA) was completed with the metabolite datasets for thoraxes, reducing all the variables to the first two principal components. Biplots were generated first, with vector labels corresponding to the metabolite. Scores plots were created to visualize time-based clustering and shifts in metabolic profiles throughout the duration of flight. Each point on the scores plot represents one individual’s metabolic profile.

## Data Availability

Data are contained within the article or Supplementary Material.

## Supplementary Materials

The following are available online at https://www.mdpi.com/article/10.3390/metabo11080511/s1, Table S1: Signal intensity of all metabolites for each individual bee measured at various flight time.
